# Giant pulmonary hamartoma

**DOI:** 10.1186/1749-8090-1-19

**Published:** 2006-08-03

**Authors:** Somshekar Ganti, Richard Milton, Les Davidson, Vladimir Anikin

**Affiliations:** 1 Department of Thoracic Surgery, St. James University Hospital, Beckett Street, Leeds LS9 7T, UK; 2 Department of Histopathology, Leeds General Infirmary, Great George Street, Leeds LS1 3EX, UK

## Abstract

Pulmonary hamartomas are usually an incidental finding and range in size from 1 cm to 8 cm in diameter in various series. We report a case of a massive pulmonary hamartoma (size 25.5 × 17.5 × 6.5 cm and weighing 1134 g) in a 61 year old male who presented with a short history of breathlessness. The tumour was arising from the medial border of the right lung and occupying most of the right chest extending in to the anterior mediastinum. The tumour was compressing the right lung and there was no evidence of infiltration into the surrounding structures. It was successfully treated by surgical resection and final histology was pulmonary hamartoma with predominantly adipose and leiomyomatous differentiation.

## Introduction

Pulmonary hamartomas, also known as mesenchymomas, can be parenchymal (80%) or endobronchial (10–20%) in location [1]. Parenchymal lesions are usually an incidental finding and range in size from 1 cm to 8 cm in diameter in various series [6]. The endobronchial tumours usually present with new onset respiratory symptoms most commonly recurrent chest infections or haemoptysis [1]. We report a case of giant hamartoma which we believe is the largest reported case in literature yet, and this was successfully resected via a median sternotomy.

## Case report

**Figure 1:**
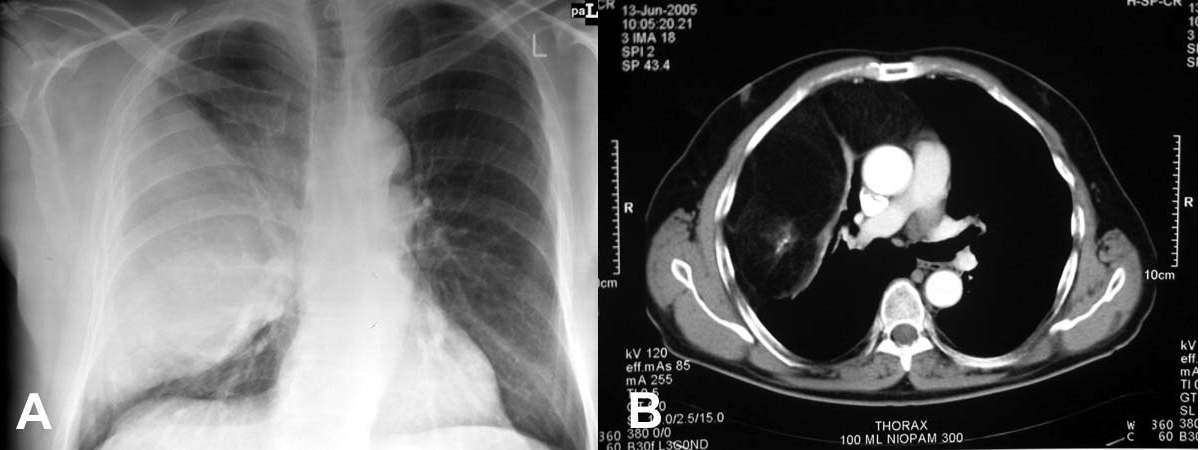
Chest radiograph showing soft tissue mass within the right hemithorax (A). CT (B) demonstrates that the mass contains low attenuation soft tissue suggestive of fat, and calcification, features in keeping with a teratoma.

A previously healthy 61-year-old man was investigated for worsening breathlessness. Chest radiography demonstrated a mass in the right chest and a subsequent CT scan revealed a large anterior mediastinal mass extending mostly into the right hemithorax and compressing the right lung. The radiological appearances were consistent with a teratoma (Fig. 1A–B). The CT scan also revealed abdominal lymphadenopathy, which on biopsy proved to be non-Hodgkin's lymphoma. The lymphoma was successfully treated with chemotherapy, without any change in the lung mass. Nevertheless, surgical excision of this mass was indicated due to its large size.

**Figure 2:**
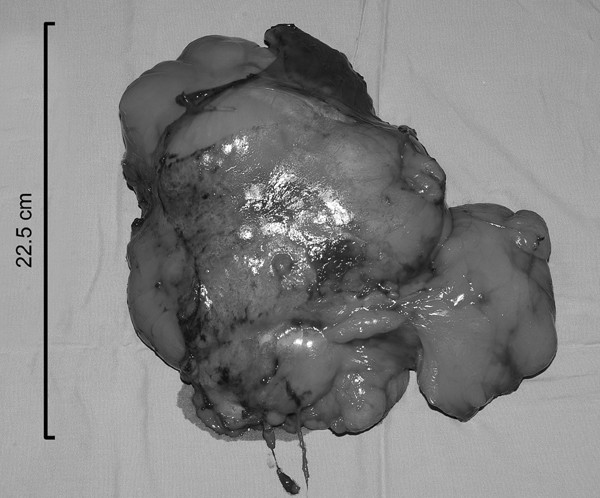
Photograph of the resected hamartoma.

The patient underwent a median sternotomy whereupon a giant fatty tumour was found arising superficially from the medial border of the right lung occupying most of the right chest and extending to the anterior mediastinum. The mass was compressing the right lung with no evidence of local invasion. The right phrenic nerve was identified and preserved, and the tumour was found to be adherent to the medial border of the lung and was easily dissected with sharp dissection and removed en bloc. The postoperative recovery was uneventful and the patient was discharged home on the seventh postoperative day. Six months postoperatively the patient remains well. The final histology of the tumour, measuring 25.5 × 17.5 × 6.5 cm, and weighing 1134 g (Fig. 2), was a pulmonary hamartoma with predominantly adipose and leiomyomatous differentiation.

## Discussion

Pulmonary hamartomas or mesenchymomas are the most common form of benign lung tumours with an incidence of between 0.025% – 0.32% according to different necropsy studies [1]. In a large series they constituted 7–14% of all coin lesions [2]. Most of these are parenchymal lesions with endobronchial lesions making up only about 10% [1]. The peak incidence is between the sixth and seventh decades with a male preponderance [3]. The parenchymal lesions are usually an incidental radiological finding of a round homogeneous opacity in the periphery, whereas endobronchial lesions are usually associated with haemoptysis and obstructive pneumonia [4]. The size of these parenchymal lesions ranges from 1–8 cm and the tumour in our case is more than 3 times that of any previously published [6,7]. The histology of the parenchymal lesions usually reveals a predominant chondroid differentiation (80%), with fibroblastic (12%), fatty (5%) and osseous (3%) differentiation making the rest [3,7]. In our case it was predominantly made of adipose and leiomyomatous differentiation. The size of the tumour and the histology make it an unusual presentation.
